# The Prevalence of Depression and Anxiety Symptoms among Overweight/Obese and Non-Overweight/Non-Obese Children/Adolescents in China: A Systematic Review and Meta-Analysis

**DOI:** 10.3390/ijerph16030340

**Published:** 2019-01-26

**Authors:** Simeng Wang, Qi Sun, Lingling Zhai, Yinglong Bai, Wei Wei, Lihong Jia

**Affiliations:** Department of Child and Adolescent Health, School of Public Health, China Medical University, Shenyang 110122, China; cwgns@163.com (S.W.); sunqi@cmu.edu.cn (Q.S.); llzhai@cmu.edu.cn (L.Z.); ylbai@cmu.edu.cn (Y.B.); wwei@cmu.edu.cn (W.W.)

**Keywords:** children, adolescents, overweight, obesity, depression symptoms, anxiety symptoms, China

## Abstract

With the dramatic growth of the Chinese economy, the number of children/adolescents with being overweight/having obesity is increasing, which has a certain impact on their psychology, such as depression and anxiety symptoms. Our purpose was to conduct a meta-analysis to assess the prevalence and odds ratios of depression and anxiety symptoms among overweight/obese children/adolescents and non-overweight/obese children/adolescents in China. As of July 2018, the three most comprehensive computerized academic databases in China have been systematically screened, namely China national knowledge infrastructure (CNKI) databases, Wanfang databases and Vip databases. The same operations are performed in PubMed and Web of Science (SCIE) databases without language restrictions. Case-control studies on prevalence of depression and anxiety symptoms in overweight/obese children/adolescents in China were analyzed. Study selection and evaluation were performed independently by three authors. Unweighted prevalence, pooled random-effects estimates of odds ratio (OR), and 95% confidence intervals (CI) were all calculated. A total of 11 eligible studies involving 17,894 subjects were included. The prevalence of depression and anxiety symptoms in overweight/obese children/adolescents was significantly higher than that in non-overweight/non-obese children/adolescents (depression: 21.73% vs. 17.96%, OR = 1.46, 95% CI: 1.14, 1.87, *p* = 0.003; anxiety: 39.80% vs. 13.99%, OR = 1.47, 95% CI: 1.21, 1.79, *p* < 0.001). Subgroup analyses conducted according to scale types showed that scale types have certain significance to evaluate the relationship between depression symptoms and overweight/obesity. The OR of depression symptoms between overweight/obese children/adolescents and non-overweight/non-obese children/adolescents was greatest on the Middle School Student Mental Health Scale (MSSMHS) was 2.06 (95% CI: 1.41, 3.02, I^2^ = 0.00%), Center for Epidemiologic Studies Depression Scale (CES-D) was 1.03 (95% CI: 0.84, 1.25, I^2^ = 0.00%), and Children’s Depression Inventory (CDI) was 1.21 (95% CI: 1.02, 1.42, I^2^ = 0.00%). We concluded that the prevalence of depression and anxiety symptoms in overweight/obese children/adolescents in China is higher than that in the non-overweight/obese children/adolescents. The results of the study indicate that the prevalence of depression and anxiety symptoms among overweight/obese children/adolescents in Chinese medical institutions should receive more attention. Physical exercise and psychological interventions should be strengthened to prevent psychological problems. However, because of some clear limitations (no clinical interview and few studies), these results should be interpreted with caution.

## 1. Introduction

Overweight and obesity among children and adolescents have become one of the most serious global public health concerns in the 21st century [1]. Over the past 30 years, the prevalence of overweightness and obesity among children and adolescents worldwide has increased at an alarming rate [1,2]. In China, the rapid economic development over the past decade has led to accelerated transformations in the Chinese diet, which has led to a significant increase in body mass index (BMI) and an increased risk of overweightness and obesity in the population [3,4,5]. There has been a proliferation of fast food restaurants and an increase in activities that lead to less physical activity, such as watching television and playing computer games [6]. In addition to reducing physical activity, television viewing also promotes the consumption of high-energy foods through incessant commercial advertisements [7]. In China’s education system, it relies almost entirely on test scores to evaluates progress, which lead to more reading, examinations, or/and homework for Chinese children and less time for physical activities [8]. In 2013, approximately 23.0% and 14.0% of Chinese boys and girls aged 2–19 years were overweight or obese [9]. Overweightness and obesity in children and adolescents can lead to developmental problems, such as poor cognitive function and altered timing of puberty [8]. Childhood obesity usually accompanies into adulthood and is associated with many serious consequences, including metabolic and cardiovascular sequelae [10,11]. In addition to the recognized physiological results, attention is increasingly being focused on determining whether mental illness may also result from obesity, such as depression and anxiety [12]. 

Depression and anxiety disorders are the most common mental disorders [13]. Depressive disorders are a variety of comprehensive disease with the characteristics of mental depression, which feature low mood, slow thinking, decreased mental activity, cognitive impairment, and physical symptoms [14]. Anxiety disorders are characterized by psychological symptoms, such as excessive worry, fear, and apprehension, as well as physical symptoms, such as fatigue, heart palpitations, and tension [15]. In different countries, the lifetime prevalence rate of depression disorders in adults is between 3.3 and 21.4%, and that of anxiety disorders is between 4.8 and 31.0% [16]. A range search retrieved for eleven reviews related to obesity and psychological factors that included depression and anxiety. A large proportion of reviews showed a link between obesity and depression disorders [17,18,19,20,21,22,23,24]. Few studies have shown moderate evidence to support a weak but positive association between anxiety and obesity [25,26]. Other studies showed that the link between anxiety and obesity is unclear [27]. Depression and anxiety are common mood disorders among adolescents [12]. Some evidence-based studies have shown that obese adolescents are more prone to mental health problems such as depression, anxiety, and poor self-esteem than non-obese adolescents [28]. Nevertheless, there are relatively few studies on the relationship between overweight/obesity and depression and anxiety symptoms in children/adolescents in China. The harm caused by childhood obesity may affect the health of the whole life, which should arouse more attention and early intervention measures.

Therefore, this meta-analysis aims to comprehensively evaluate the individual studies of depression and anxiety symptoms in overweight/obese children/adolescents in China, and to assess the prevalence and odds ratio (OR) of depression and anxiety symptoms in overweight/obese children/adolescents in China.

## 2. Materials and Methods

### 2.1. Literature Search

Our meta-analysis was conducted in accordance with Preferred Reporting Items for Systematic Reviews and Meta-Analyses (PRISMA) guidelines (http://www.prismastatement.org/). This study systematically searched the literature on the prevalence of depression and anxiety symptoms in overweight/obese children/adolescents in China. As of July 2018, the three most comprehensive computerized academic databases in China have been systematically screened, namely China national knowledge infrastructure (CNKI) databases, Wanfang databases, and Vip databases. We used “depression or depressive disorders or depressive symptoms” and “anxiety or anxiety disorder or anxiety symptoms” combined with “children or adolescents” and “overweight or obesity” as search topics for article titles, abstracts, and keywords. The reference lists of relevant literatures were screened.

In order to expand the scope of the search, the same operations are performed in PubMed and Web of Science (SCIE) databases without language restrictions. The search strategy was: (obesity [MeSH Terms] OR overweight [MeSH Terms] OR obesity [Title/Abstract] OR overweight [Title/Abstract]) AND (depression [MeSH Terms] OR depressive disorder [MeSH Terms] OR depression [Title/Abstract] OR depressive disorder [Title/Abstract] OR depressive symptoms [Title/Abstract] OR anxiety [MeSH Terms] OR anxiety disorder [MeSH Terms] OR anxiety [Title/Abstract] OR anxiety disorder [Title/Abstract] OR anxiety symptoms [Title/Abstract]) AND (children [MeSH Terms] OR adolescent [MeSH Terms] OR children [Title/Abstract] OR adolescent [Title/Abstract]) AND (China [MeSH Terms] OR China [Title/Abstract]).

The screening of the abstracts/titles and full-text articles were done twice independently by three authors (S.W., Q.S., and L.Z.) to reduce the bias and errors of reviewers.

### 2.2. Inclusion and Exclusion Criteria

We included all the following studies: (1) the subjects were children/adolescents under 18 years old; (2) qualified case-control studies, including overweight/obese and non-overweight/non-obese children/adolescents; (3) studies were established in China; (4) studies that defined overweight/obesity categories based on body mass index (BMI) were included [29], where BMI was calculated by dividing body weight (kg) by the height squared (m^2^); (5) depression and anxiety symptoms in the overweight/obese children/adolescents and the non-overweight/non-obese children/adolescents were identified by self-reported questionnaires, and previous studies have established their reliability as a measurement indicator of depression and anxiety symptoms; and (6) the prevalence of depression and anxiety symptoms in the overweight/obese children/adolescents and the non-overweight/non-obese children/adolescents have been reported. We excluded the following studies: (1) the studies only included overweight/obese children and adolescents; (2) the overweight/obese children/adolescents and the non-overweight/non-obese children/adolescents had no strict criteria. Eligibility judgment and data extraction were independently recorded and performed by the two authors (L.Z. and Y.B.) in a standardized manner. L.Z. and Y.B. used a pretested standardized form to extract information from each eligible study including participants and cluster demographics, regional distribution, study methodology, and outcome data. Any differences with them were settled through discussion and the participation of another author (L.J.).

### 2.3. Quality Assessment

Although existing checklists and quality assessment scales are controversial in observational studies [30], the Newcastle–Ottawa scale was used to assess the quality of observational and non-randomized studies [31]. The instrument evaluated observational studies according to the following three criteria: case selection, comparability of the study group and assessment of results or exposure. Three categories were defined: the study was regarded to have high quality (low risk of bias) if the score was more than 6, studies that scored 1 or zero were considered as having low quality (high risk of bias), studies that scored 2–6 were categorized as having medium quality (moderate risk of bias). Any controversies with raters (L.B. and W.W.) were determined by another author (L.J).

### 2.4. Meta-Analysis

#### 2.4.1. Assessment of Overall Effect Size

The effect size of OR is defined as the ratio of odds (odds = probability/(1 − probability)) of depression and anxiety symptoms between the overweight/obese children/adolescents and the non-overweight/non-obese children/adolescents. An OR greater than 1 indicates that the overweight/obese children/adolescents were more prone to depression/anxiety symptoms than the non-overweight/non-obese children/adolescents, while an OR less than 1 indicates that the overweight/obese children/adolescents were less likely to be depressed/anxious. The standard method of inverse variance weighting was used to calculate the pooled random-effects estimates of OR and 95% confidence intervals (CI), so as to ensure that larger and more accurate estimates were given relatively more weighting, and non-weighted prevalence rates were also calculated. The random effects model was used because it involves the assumption of statistical heterogeneity between studies [32,33]. Overall effects were analyzed by Stata v11.0 statistical software (STATA Corporation, College Station, TX, USA).

#### 2.4.2. Assessment of Heterogeneity

Heterogeneity was evaluated using Q statistics and I^2^ statistics. The Q statistic is used to assess whether differences in results are only compatible with chance. If the *p*-value of the Q statistic is greater than 0.05, the heterogeneity is not significant [34], but the Q statistic is sensitive to the number of studies [35]. In order to supplement the Q statistic, the I^2^ statistic was calculated and reported. The I^2^ statistic denotes the variance among studies as a proportion of the total variance because I^2^ is insensitive to the number of studies [35]. Larger values of I^2^ show increasing heterogeneity. No heterogeneity was observed when I^2^ was 0%, low heterogeneity was 25%, medium heterogeneity was 50%, and high heterogeneity was 75% [36].

#### 2.4.3. Meta-Regression and Subgroup Analyses

We conducted a random-effects meta-regression to determine the degree of heterogeneity due to study-specific covariates, namely age in our model. When the hypothesis of homogeneity was rejected by the Q statistic and I^2^ statistic, subgroup analysis was conducted to explore possible regulatory factors of heterogeneity [37]. In our study, we performed a subgroup analysis of regulatory factors including age groups, scale types and regional distribution.

#### 2.4.4. Assessment of Publication Bias

Some authors have argued that visual interpretation of funnel plots is too subjective and cannot be useful [38]. Then, Begg’s test and Egger’s test were further used to test its existence more objectively (implemented in Stata v11.0) [39,40]. 

## 3. Results

### 3.1. Study Selection

**Figure 1:**
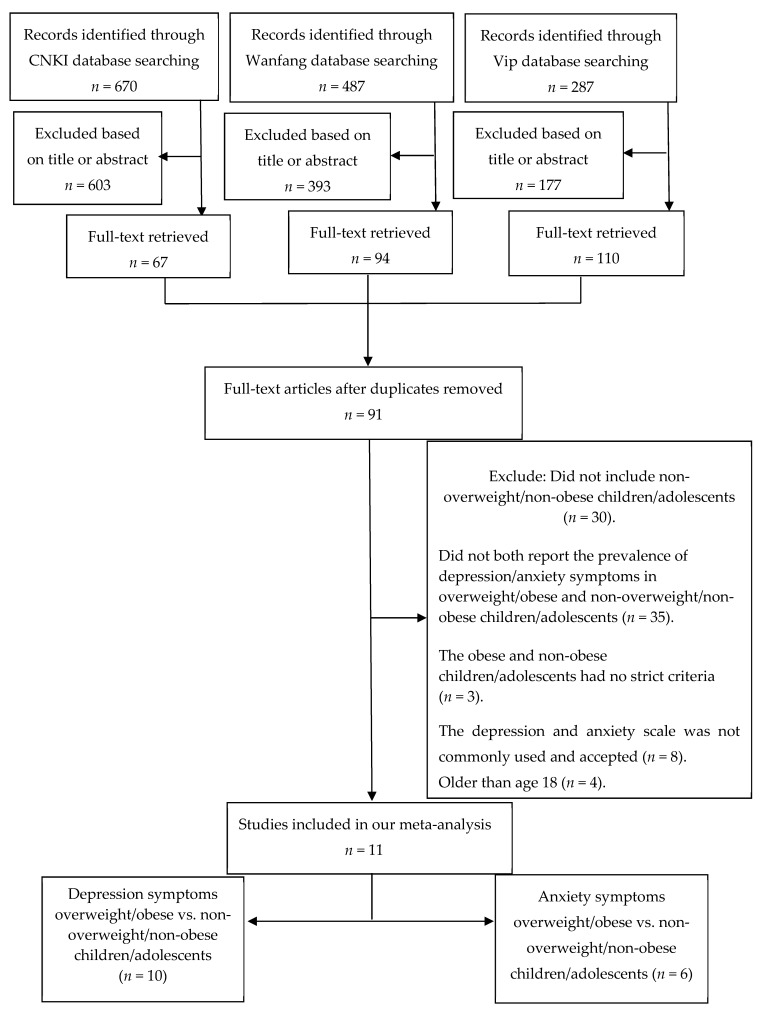
The selection process of the study.

**Figure 2:**
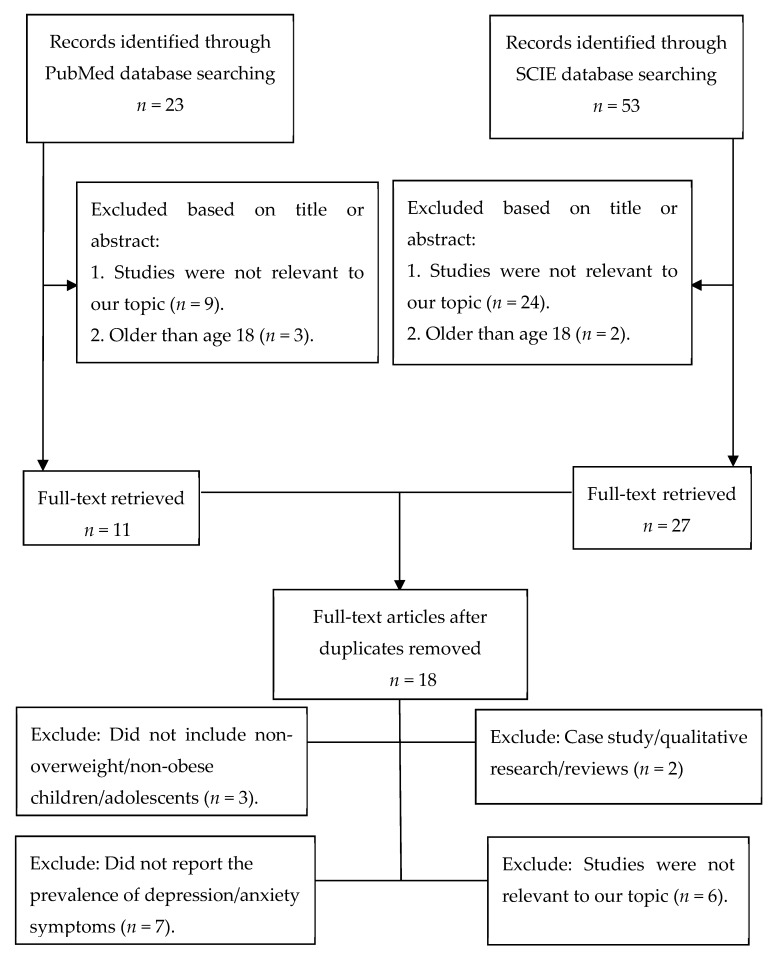
The selection process of the study (international databases).

A flowchart showing the inclusion and exclusion process was given. As shown in Figure 1, we screened out potentially qualified articles through the CNKI databases (*n* = 670), Wanfang databases (*n* = 487), and Vip databases (*n* = 287). The three authors (S.W., Q.S., and L.Z.) studied the titles and abstracts of these potentially eligible papers, and the full-text articles without duplicates (*n* = 91) were selected for further study. Based on the full-text of these 91 studies, we finally selected 11 studies for this meta-analysis [41,42,43,44,45,46,47,48,49,50,51]. The most significant reasons for exclusion were: the non-overweight/non-obese children/adolescents was not included (*n* = 30), and the prevalence of depression/anxiety symptoms in both the overweight/obese children/adolescents and the non-overweight/non-obese children/adolescents was not reported (*n* = 35). Other reasons included subjects’ age, assessment of depression and anxiety symptoms, and the composition of control group. To extend the scope of the search, we also searched two international databases, PubMed and SCIE (as shown in Figure 2). Nevertheless, through two international database searches, we did not find any literature that met our inclusion and exclusion criteria.

### 3.2. Characteristics of Included Studies

Eleven studies involving 17,894 subjects produced two subgroups: (1) depression symptoms in overweight/obese and non-overweight/non-obese children/adolescents (*n* = 10), and (2) anxiety symptoms in overweight/obese and non-overweight/non-obese children/adolescents (*n* = 6) (Figure 1). Study characteristics are showed in Table 1. This meta-analysis included 7 journal papers, 1 doctoral thesis, and 3 master’s theses published from 2004 to 2015. Most of the studies showed significant differences between overweight/obesity and depression or anxiety symptoms [41,42,43,44,45,46,47,48,49,50], except for one study [51].

### 3.3. Risk of Bias Assessment

The study quality score for each Newcastle–Ottawa standard are shown in Table 2, where higher scores reflect the better study quality. The average score of all studies was more than 5 points. Five studies were rated as high quality. Six studies were rated as medium quality. 

### 3.4. Prevalence Rates of Depression and Anxiety Symptoms in Overweight/Obese Children/Adolescents

As shown in Table 3, the overall prevalence of depression and anxiety symptoms in overweight/obese children/adolescents was 21.73% and 39.80%, respectively, and that in non-overweight/obese children/adolescents was 17.96% and 13.99%, respectively. The prevalence of depression (*p* < 0.01) and anxiety symptoms (*p* < 0.001) in overweight/obese children/adolescents was significantly higher than that in non-overweight/non-obese children/adolescents. Based on the Middle School Student Mental Health Scale (MSSMHS) (*p* < 0.001) and Children’s Depression Inventory (CDI) (*p* < 0.05), the prevalence of depression symptoms in overweight/obese children/adolescents was higher than that in non-overweight/obese children/adolescents.

### 3.5. Odds Ratios of Depression and Anxiety Symptoms in Overweight/Obese Children/Adolescents

**Figure 3:**
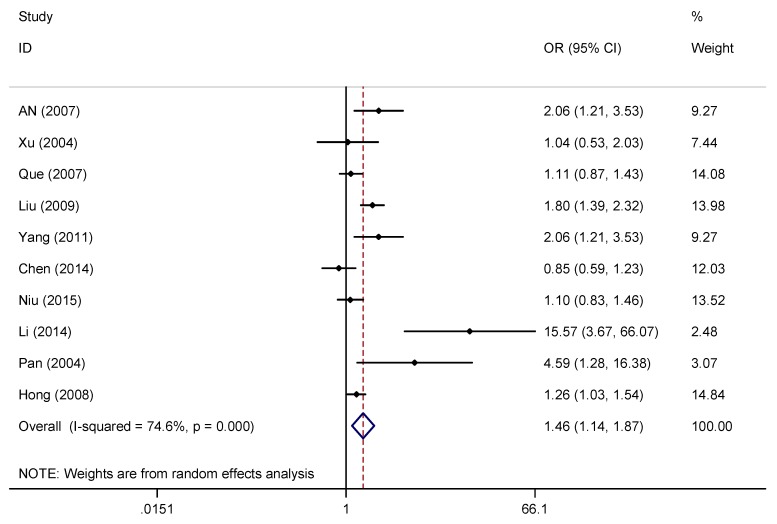
Forest plot for the meta-analysis of depression symptoms in children/adolescents with and without overweight/obesity.

**Figure 4:**
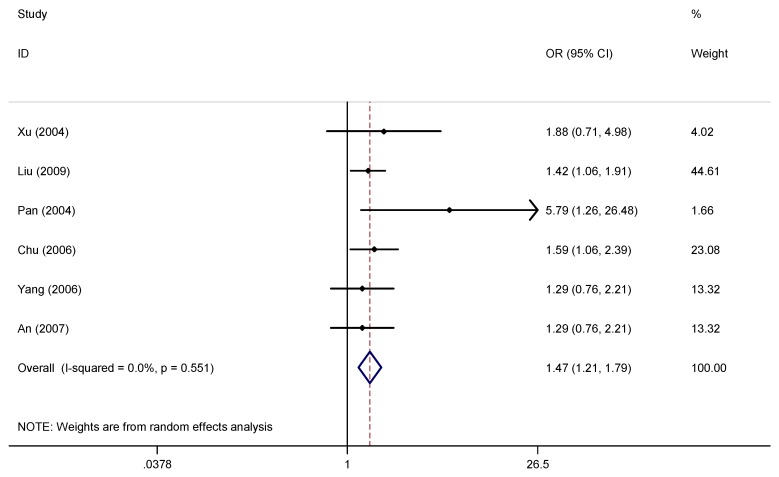
Forest plot for the meta-analysis of anxiety symptoms in children/adolescents with and without overweight/obesity.

A pooled random effects meta-analysis used data from 11 studies that estimated the levels of depression and anxiety symptoms in overweight/obese children/adolescents. This analysis included data for 3,038 children/adolescents with overweight/obesity and 14,856 without overweight/obesity. As shown in Figure 3 and Figure 4, the OR of depression symptoms was associated with a 1.46-fold increased risk of overweight/obese children/adolescents when compared with non-overweight/non-obese children/adolescents (OR = 1.46, 95% CI: 1.14, 1.87; *p* = 0.003), and the OR of anxiety symptoms was also more than 1.53 times as high in overweight/obese children/adolescents compared with non-overweight/non-obese children/adolescents (OR = 1.47, 95% CI: 1.21, 1.79; *p* < 0.001). However, the heterogeneity analysis of the effect sizes of depression symptoms (Q = 35.41, *p* < 0.001; I^2^ = 74.6%) indicated that our meta-analysis was highly heterogeneous.

### 3.6. Meta-Regression

**Figure 5:**
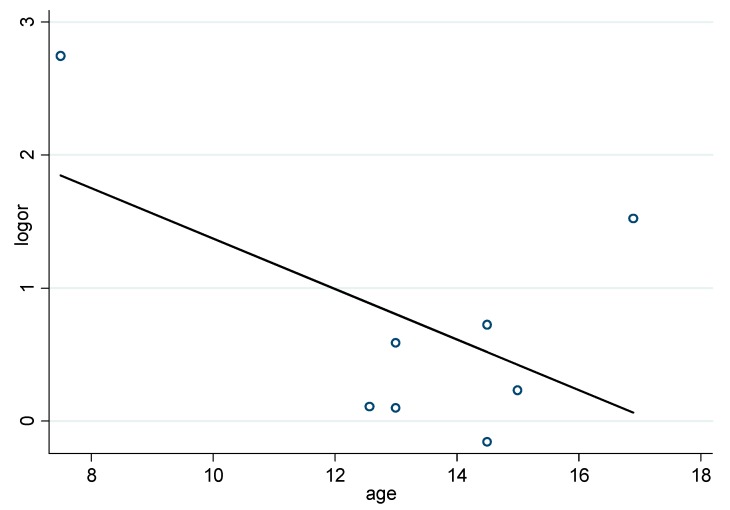
Results of the meta-regression indicated that the OR did not vary significantly with age.

As shown in Figure 5, unexpected results of the meta-regression did not provide sufficient evidence to convince us that age was a covariate leading to heterogeneity (*p* = 0.298).

### 3.7. Subgroup Analyses

**Figure 6:**
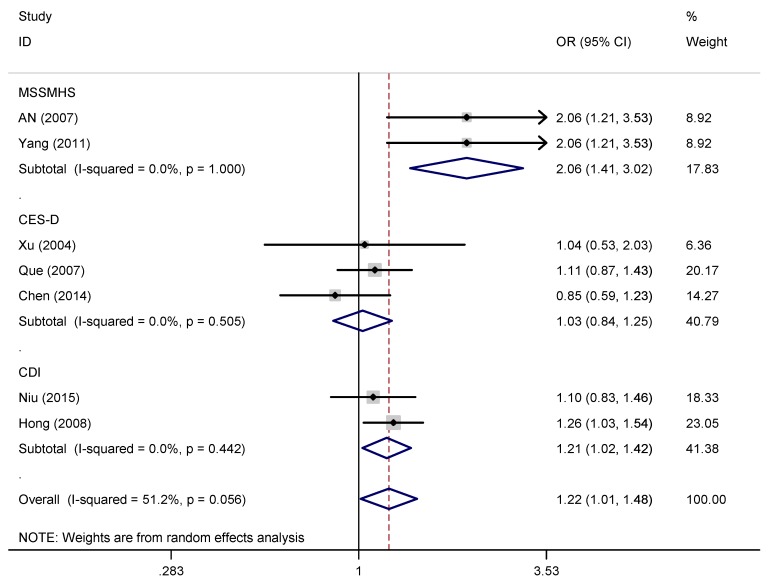
Subgroup analysis by scale types.

**Figure 7:**
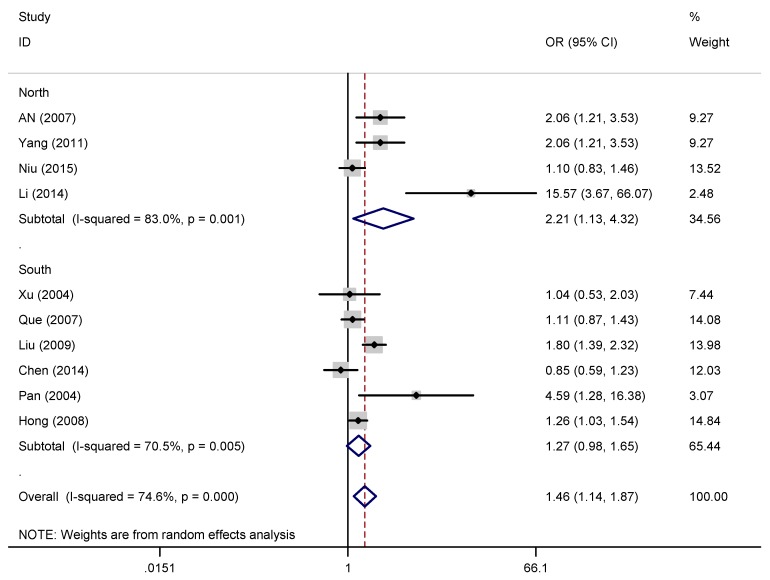
Subgroup analysis by regional distribution.

**Figure 8:**
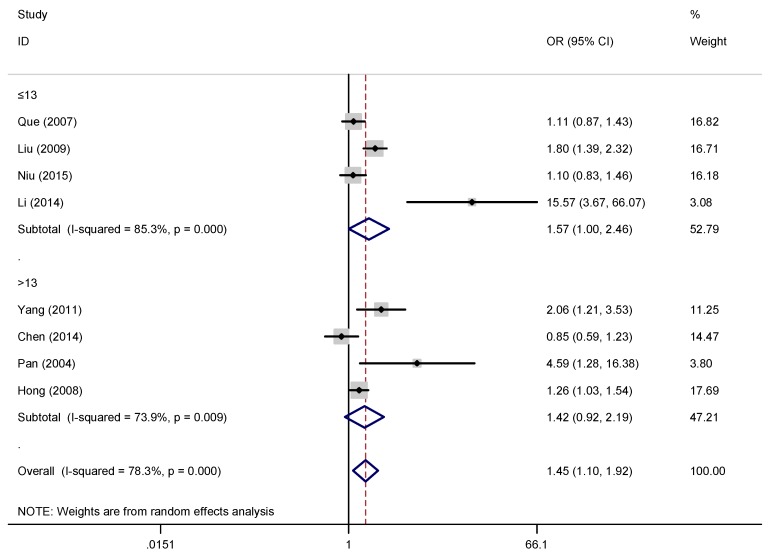
Subgroup analysis by age groups.

Subgroup analyses (Table 4 and Figure 6, Figure 7 and Figure 8) conducted by scale types showed that the ORs of depression between overweight/obese children/adolescents and non-overweight/non-obese children/adolescents was greatest on MSSMHS scale was 2.06 (95% CI: 1.41, 3.02, I^2^ = 0.00%), CES-D scale was 1.03 (95% CI: 0.84, 1.25, I^2^ = 0.00%), and CDI scale was 1.21 (95% CI: 1.02, 1.42, I^2^ = 0.00%). However, subgroup comparison of children/adolescents with depression symptoms in different regional distribution and ages groups showed no significance. 

### 3.8. Publication Bias

**Figure 9:**
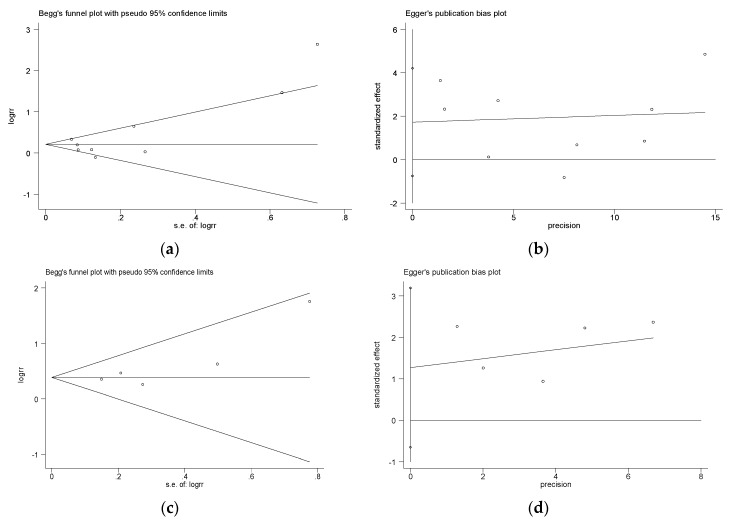
Publication bias in depression and anxiety symptoms.

The Begg’s test and Egger’s test further showed that there was no publication bias in depression symptoms (Begg’s test, *p* = 0.325; Figure 9a; Egger’s test, *p* = 0.148; Figure 9b) and anxiety symptoms (Begg’s test, *p* = 0.348; Figure 9c; Egger’s test, *p* = 0.141; Figure 9d) in our meta-analysis.

## 4. Discussion

With the dramatic growth of the Chinese economy, the number of overweight/obese children/adolescents is increasing, which has a certain impact on their psychology, such as depression and anxiety symptoms. The harm caused by childhood obesity may affect the health of their whole life, which should arouse more attention and early intervention measures. To our knowledge, this is the first meta-analysis reporting depression and anxiety symptoms between overweight/obese children/adolescents and non-overweight/non-obese children/adolescents in China. We applied a systematic approach to the literature search, including local journal websites in China. The use of the quality assessment criteria standardized the evaluation method and reduced the risk of subjectivity. The Newcastle–Ottawa Scale was used to evaluate the quality of the study. We identified only five studies of high quality. There are some methodological defects in quality assessment, which may weaken the internal validity.

This meta-analysis included 11 case-control studies with a total of 17,894 participants, from 2004 to 2015, seven journal papers, one doctoral thesis, and three master’s theses were published. The results showed that the overall prevalence of depression and anxiety symptoms among overweight/obese children/adolescents was 21.73% and 39.80%, respectively, and that among non-overweight/obese children/adolescents was 17.96% and 13.99%, respectively, indicating that depression and anxiety symptoms also did coexist among overweight/obese children/adolescents in China [41,42,44,45,50], similar to this situation in foreign countries [52,53]. Because of the particularity of the education model in China, parents pay more attention to their children’s academic performance and often ignore their psychological activities. This phenomenon leads to children and adolescents having high learning pressure and little physical activity, which is one of the reasons why obese children are increasingly showing depressive and anxiety disorders. This situation should be noted because comorbid depression and anxiety tend to have more severe symptoms, worse outcomes, and greater use of medical resources than people with a single disorder. However, findings on the association between overweight/obese children/adolescents and depression/anxiety symptoms are mixed. Some studies also found that obese children have a slightly increased risk of depression [22,23,24,54,55] and anxiety symptoms [55] compared with non-obese children. Several studies found that obese children/adolescents have fewer psychological problems (depression/anxiety symptoms) than their non-obese peers [20,26,27,56,57]. Robert et al. used prospective data to examine the relationship between anxiety disorders and weight among adolescents. They reported that neither obesity nor being overweight increased the risk of future of anxiety disorders [58]. Therefore, the causal relationship between obesity/overweight and depression/anxiety symptoms cannot be inferred, and future etiological studies are recommended.

It is not enough to report the prevalence of depression and anxiety symptoms among overweight/obese children/adolescents in China. It is of great significance that this study included comparable non-overweight/non-obese group, such that the level of depression and anxiety symptoms in Chinese overweight/obese children/adolescents can be measured reliably and accurately. The level of depression (OR = 1.46, 95% CI: 1.14, 1.87) and anxiety symptoms (OR = 1.47, 95% CI: 1.21, 1.79) in overweight/obese children/adolescents was significantly higher than that in non-overweight/obese children/adolescents.

Heterogeneity and quality of study were assessed in this meta-analysis. First, we conducted a random-effects meta-regression to determine the degree of heterogeneity due to study-specific covariates, namely age in our model. Nevertheless, unexpected results of the meta-regression did not provide sufficient evidence to convince us that age was a covariate leading to heterogeneity. The strict inclusion criteria, random effects models, and subgroup analyses were used to control and reduce heterogeneity. We have conducted subgroup analysis according to different age groups, the ORs of depression symptoms in obese children/adolescents and non-obese children were not statistically significant for subgroups with different age groups. Thus, age may not be an important factor in the relationship between overweight/obese children/adolescents and depression and anxiety symptoms. However, due to the small number of studies and high heterogeneity, one should be very careful with the interpretation. Further studies should be conducted to verify whether age has an effect on depression symptoms in overweight/obesity children/adolescents. Second, subgroup analysis based on the types of scale have a certain significance in assessing the relationship between depression symptoms and overweight/obesity. In the subgroup analysis, we excluded three studies because they used different scales to limit the grouping and the heterogeneity was significantly lower. We can infer that scale types play an important role in analyzing the relationship between depression symptoms and overweight/obesity. We suggest evaluating the relationship between overweight/obesity and depression symptoms with one or more types of scales to ensure homogeneity. Third, when subgroup analysis was conducted according to different regional distribution, subgroup comparison of children/adolescents with depression symptoms in different regional distribution showed no significance. The heterogeneity was still relatively higher. Thus, regional distribution had little impact on depression and anxiety symptoms in children/adolescents with overweight/obesity. Due to the small number of studies, and it was not clearly stated in the studies, subgroup comparison of depression symptoms in children/adolescents of different gender were not analyzed. 

This study has some limitations. First, all the studies focused on the population of China. In China, the available literature on the relationship between overweight/obesity and depression/anxiety symptoms in children/adolescents is very limited. Although our electronic database search was as comprehensive as possible, without the limitations of language and year of publication, grey literature and other published studies may not be recognized because the original language of the Chinese language included is not English. This may exclude relevant qualified studies from our systematic search. Second, the total number of studies included in our meta-analysis was relatively small. Consequently, our results need to be confirmed in more diverse samples with different racial and ethnic backgrounds and gender balances before we can generalize the estimates. Third, the BMI is discussed as being a difficult indicator for overweight/obesity in children/adolescents. Using the percentiles that better reflect the age group and the adequate weight might be a more reliable and valid measure. However, this method is rarely used in China. In our meta-analysis, only three studies included elementary school students. The proportion of children is relatively small. This is also a limitation of our study. Fourth, the cut point used for the CES-D of 16 was derived in adults and has poor specificity in children and adolescents. Cut scores for children and adolescents have been reported at 20 and even as high as 24. Thus, the prevalence of depression symptoms using the CES-D would likely be an overestimate. Fifth, the measures used in the component studies were all screening instruments. The cut points on these instruments indicate a probability of disorder that needs then to be confirmed by a clinical assessment. We will further use clinical interviews and self-assessment questionnaires as the diagnostic criteria for depression and anxiety symptoms. Finally, due to insufficient data, it was not possible to assess the potential interaction of this bidirectional association. Further study is needed to determine whether these relationships can be replicated in a larger, multiethnic, and socioeconomically diverse group of overweight and normal weight adolescent population.

## 5. Conclusions

We concluded that the prevalence of depression and anxiety symptoms in overweight/obese children/adolescents in China was higher than that in the non-overweight/obese group. The findings support that screening for depression and anxiety symptoms in overweight/obese children/adolescents should be a preliminary recommendation for effective intervention in overweight/obese children/adolescents, and the prevalence of depression and anxiety symptoms among overweight/obese children/adolescents in China’s medical institutions should receive more attention. Physical exercise and psychological interventions should be strengthened to prevent psychological problems. However, because of some clear limitations (no clinical interview and few studies), these results should be interpreted with caution.

## Figures and Tables

**Table 1:** Characteristics of inclusion in the study.

Author and Years	Depression/Anxiety Symptoms	N1/N2	Region	Sample	Mean Age	Age Range	Depression/Anxiety Scale and Cut-Off	Mean ± SD (N1,N2)	Depression/Anxiety Symptoms (%)
An et al. [41], 2007	depression	136/726	North	Middle school students	- -	- -	self-report (MSSMHS factor score ≥ 3)		15.4/8.2
	anxiety	136/726			- -	- -	self-report (MSSMHS factor score ≥ 3)		14.0/11.2
Xu et al. [42], 2004	depression	97/100	South	Secondary school students	- -	- -	self-report (CES-D total score ≥ 16)		22.7/22.0
	anxiety	97/100			- -	- -	self-report (SAS total score ≥ 40)		12.4/7.0
Pan et al. [45], 2004	depression	184/184	South	High school students	16.90 ± 1.47 16.90 ± 1.47	- -	self-report (SCL-90 factor score ≥ 3)	1.74 ± 0.73, 1.61 ± 0 60	7.1/1.6
	anxiety	184/184			16.90 ± 1.47 16.90 ± 1.47	- -	self-report (SCL-90 factor score ≥ 3)	1.66 ± 0.72, 1.50 ± 0.51	6.0/1.1
Que [43], 2007	depression	359/1412	South	Middle school students	12.57 ± 0.708 12.57 ± 0.708	- -	self-report (CES-D total score ≥ 16)		32.0/29.7
Liu [44], 2009	depression	300/1202	South	Middle school students	13 13	12–14 12–14	self-report (SDS total score ≥ 53)	51.74 ± 8.97, 50.83 ± 8.73	50.3/36.0
	anxiety	301/1238			13 13	12–14 12–14	self-report (SAS total score ≥ 51)	45.00 ± 9.17, 44.22 ± 8.73	25.9/19.7
Hong et al. [46], 2008	depression	756/6405	South	Middle school students	15 15	12–18 12–18	self-report (CDI standard score ≥ 19)	12.25 ± 7.60, 11.5 ± 7.10	17.6/14.5
Chen et al. [47], 2014	depression	160/941	South	Middle school students	14.5 14.5	11–18 11–18	self-report (CES-D total score ≥ 16)		28.8/32.1
Niu et al. [48], 2015	depression	470/1970	North	Primary and secondary school students	13 13	9–17 9–17	self-report (CDI total score ≥ 19)		15.1/13.9
Li [51], 2014	depression	260/260	North	Elementary school students	7.5 7.5	3–12 3–12	self-report (CBCL factor score ≥ 3)		10.8/0.8
Chu [49], 2006	anxiety	179/894	North	Elementary school students	9.5 9.5	7–12 7–12	self-report (PHCSS factor score < 8 or >14)		20.7/14.1
Yang [50], 2006	depression	136/726	North	Middle school students	14.5 14.5	12–17 12–17	self-report (MSSMHS factor score ≥ 3)		15.4/8.1
	anxiety	136/726			14.5 14.5	12–17 12–17	self-report (MSSMHS factor score ≥ 3)		14.0/11.2

Abbreviations: MSSMHS, Middle School Student Mental Health Scale; CES-D, Center for Epidemiologic Studies Depression Scale; SAS, Self-rating Anxiety Scale; SDS, Self-rating Depression Scale; CDI, Children’s Depression Inventory; SCL-90-D, Symptom Checklist 90-Depression; SCL-90-A, Symptom Checklist 90-Anxiety; CBCL, Child Behavior Checklist; PHCSS, Children’s Self-Concept Scale; N1, overweight/obese children/adolescents; N2, non-overweight/non-obese children/adolescents.

**Table 2:** Assessment of Study Quality.

Studies	Quality Indicators from Newcastle–Ottawa Scale									
	1	2	3	4	5A	5B	6	7	8	Total Score
An et al. [41], 2007	Yes	Yes	No	Yes	Yes	No	No	Yes	No	5
Xu et al. [42], 2004	Yes	Yes	Yes	Yes	Yes	Yes	No	Yes	No	7
Pan et al. [45], 2004	Yes	Yes	Yes	Yes	Yes	Yes	No	Yes	No	7
Que [43], 2007	Yes	Yes	Yes	Yes	Yes	Yes	No	Yes	No	7
Liu [44], 2009	Yes	Yes	Yes	Yes	Yes	Yes	No	Yes	No	7
Hong et al. [46], 2008	Yes	Yes	No	Yes	Yes	Yes	No	Yes	No	6
Chen et al. [47], 2014	Yes	Yes	No	Yes	Yes	Yes	No	Yes	No	6
Niu et al. [48], 2015	Yes	Yes	No	Yes	Yes	Yes	No	Yes	No	6
Li [51], 2014	Yes	Yes	No	Yes	Yes	Yes	No	Yes	No	6
Chu [49], 2006	Yes	Yes	Yes	Yes	Yes	Yes	No	Yes	No	7
Yang [50], 2006	Yes	Yes	No	Yes	Yes	Yes	No	Yes	No	6

Abbreviations: 1, indicates cases independently validated; 2, cases are representative of population; 3, potential confounding adjustment; 4, the source of the control is described; 5A, study controls for age; 5B, study controls for additional factor(s); 6, depression/anxiety symptoms was determined by blinded structured interview or secure record; 7, the same method was used to determine cases and controls; and 8, nonresponse rates are the same for cases and controls.

**Table 3:** Unadjusted prevalence of depression and anxiety symptoms in children/adolescents with and without overweight/obesity.

Variables	No. of Studies	No. of Subjects	Overweight/Obese Subjects (%)	Non-Overweight/Non-Obese Subjects (%)
**Depression symptoms** (All)	10	16784	21.73 **	17.96
**Scale type**				
MSSMHS	2	1724	24.42 ***	8.12
CES-D	3	3069	29.71	30.33
CDI	2	9601	16.64 *	14.34
**Anxiety symptoms** (All)	6	4901	39.80 ***	13.99
**Scale type**				
MSSMHS	2	1724	13.97	11.16
SAS	2	1736	22.61	18.76

Abbreviations: MSSMHS, Middle School Student Mental Health Scale; CES-D, Center for Epidemiologic Studies Depression Scale; CDI, Children’s Depression Inventory; SAS, Self-Rating Anxiety Scale. Note: * *p* < 0.05, ** *p* < 0.01, and ***p < 0.001 compared with a non-overweight/non-obese children/adolescents.

**Table 4:** Subgroup analyses of odds ratios of depression symptoms in children/adolescents with and without overweight/obesity.

Subgroups	No. of Studies	No. of Subjects	OR	95% CI	Q	I^2^ (%)	p *
Depression							
Scale type							0.01
MSSMHS	2	1724	2.06	1.41–3.02	0.00	0.00	
CES-D	3	3069	1.03	0.84–1.25	1.37	0.00	
CDI	2	9601	1.21	1.02–1.42	0.59	0.00	
Regional distribution							0.37
North	4	4532	2.21	1.13–4.32	17.64 ***	83.00	
South	6	12100	1.27	0.98–1.65	16.95 **	70.50	
Age groups							0.55
≤13	4	6233	1.57	1.00–2.46	20.39	85.30	
>13	4	9492	1.41	0.92–2.19	11.49	73.90	

Abbreviations: MSSMHS, Middle School Student Mental Health Scale; CES-D, Center for Epidemiologic Studies Depression Scale; CDI, Children’s Depression Inventory; SAS, Self-rating Anxiety Scale. Note: ** *p* < 0.01, *** *p* < 0.001. p * of comparison between these subgroups.
